# Effects of Noradrenergic Stimulation Upon Context-Related Extinction Learning Performance and BOLD Activation in Hippocampus and Prefrontal Cortex Differ Between Participants Showing and Not Showing Renewal

**DOI:** 10.3389/fnbeh.2019.00078

**Published:** 2019-04-24

**Authors:** Silke Lissek, Anne Klass, Martin Tegenthoff

**Affiliations:** Department of Neurology, BG University Hospital Bergmannsheil, Ruhr-University Bochum, Bochum, Germany

**Keywords:** extinction, renewal, hippocampus, prefrontal cortex, noradrenaline, atomoxetine

## Abstract

While the neural structures mediating context-related renewal of extinction are well established, the neurotransmitter systems processing renewal remain elusive. Noradrenergic stimulation before extinction improved learning, but did not alter renewal. Since context processing already during initial conditioning can influence renewal, in this fMRI study we investigated how noradrenergic stimulation by a single dose of atomoxetine (ATO) before initial acquisition of a context-related predictive-learning task affects subsequent learning and renewal in humans. ATO participants showing contextual renewal (REN) exhibited a selective extinction learning deficit compared to placebo (PLAC) and ATO participants lacking renewal (ATO NoREN), probably owing to formation of more stable associations during acquisition. New learning and retrieval during the extinction phase as well as initial acquisition were unimpaired. In ATO REN, higher activation in right inferior frontal gyrus (iFG) during acquisition may have supported the formation of more stable associations, while reduced activation in hippocampus and left iFG during extinction was associated with impaired context encoding and response inhibition. During recall, ATO REN showed reduced overall context-dependent renewal associated with higher activation in medial PFC and right hippocampus. The results demonstrate the importance of noradrenergic processing in inferior frontal cortex and hippocampus for human extinction learning, but not necessarily initial conditioning. Since an identical atomoxetine treatment evoked diverging blood-oxygen level dependent (BOLD) activation patterns in REN and NoREN participants, the effect is presumably related to the participants’ preferred processing strategies that may have recruited differentially interconnected networks in which noradrenergic stimulation produced diverging consequences. In the ATO REN group, probably an additive effect of their preferred processing strategy, which pre-activated the noradrenergic system, and the experimental treatment caused a shift beyond the optimal working range of the noradrenergic system, thus modulating BOLD activation in a way that impaired extinction learning and recall.

## Introduction

The phenomenon of renewal, next to reinstatement and spontaneous recovery, delivers evidence that extinction constitutes not forgetting, but rather inhibition of a previously acquired response (Bouton, 2002; Phelps et al., 2004). Renewal is defined as the recovery of a previously extinguished response if the contexts of extinction and retrieval differ (Bouton and Bolles, 1979) and thus underlines the context-dependency of extinction learning. Research on renewal can provide important insights in the mechanisms that operate on extinction processes and that may prevent successful and lasting extinction in exposure therapies.

A recent study demonstrated that renewal during extinction recall is mediated by hippocampus and ventromedial PFC (vmPFC) regions (Lissek et al., 2013): participants who showed renewal during extinction recall exhibited significantly more prominent hippocampal activation during extinction learning than participants who did not show renewal, and higher vmPFC activation during retrieval proper. These findings corroborate previous results that found hippocampus and vmPFC participating in context processing (Kalisch et al., 2006; Milad et al., 2007). Another region repeatedly found involved in extinction learning and recall is inferior frontal gyrus (iFG) with Brodmann areas BA 44, 45, and 47 (Lissek et al., 2015a,b; Klass et al., 2017). This region participates in response inhibition (Konishi et al., 1999), a process that is necessary for operant/instrumental extinction learning (Bouton et al., 2016). In particular right-hemispheric iFG has been implicated in processing response inhibition (Garavan et al., 1999; Hampshire et al., 2010), while a lesion study showed that also left iFG is critical for response inhibition (Swick et al., 2008).

In contrast to the extinction learning phase, the role of the acquisition phase for later renewal has been widely neglected. This neglect may be due to an assumption that renewal of extinguished associations during extinction recall is predominantly based on context processing during extinction learning, which again is engendered by the surprising change in outcome during extinction (Bouton, 2004; Rosas and Callejas-Aguilera, 2006) that directs attention to the context. This view assigns a central role to attention for extinction context processing and thus for evoking renewal (Darby and Pearce, 1995; Rosas and Bouton, 1997; Uengoer and Lachnit, 2012) and, on the flipside, assumes that context processing during acquisition is negligible and does not drive renewal. However, there is evidence that context processing can already occur during acquisition (Effting and Kindt, 2007) and that it can be associated with later renewal (Lissek et al., 2016). Hippocampal activation during acquisition in response to presentation of context and cue, suggesting processing of the context, was found only in participants who later showed renewal, but not in those who did not – indicating that participants with a propensity for renewal used particular encoding strategies during acquisition that encompassed the processing of context stimuli even though they were irrelevant for the current task phase.

It is highly probable that heightened attention is a key ingredient in those encoding strategies, therefore noradrenergic mechanisms may play a role, due to noradrenaline (NA) involvement in processing and control of attention (Selden et al., 1990). Animal and human studies suggest that NA is involved in directing attention toward relevant, salient information (Berridge and Waterhouse, 2003) and in cognitive flexibility, as required in attentional set-shifting (Kehagia et al., 2010). Processes of sustained and flexible attention in prefrontal cortex are importantly modulated by NA. The role of NA for extinction learning in general and in specific brain regions such as hippocampus and prefrontal regions has been demonstrated in many animal studies (e.g., Mingote et al., 2004; Rosa et al., 2013; Chai et al., 2014). The selective NA reuptake inhibitor atomoxetine was found to enhance extinction learning in rats (Janak and Corbit, 2011) and humans (Lissek et al., 2015a), and thus constitutes a promising candidate for modulation of processing also during acquisition. In a previous study, we administered a single dose of atomoxetine prior to extinction learning (Lissek et al., 2015a) in order to modulate attentional processing. Compared to placebo, the drug enhanced extinction of previously acquired stimulus–outcome associations, regardless of whether extinction occurred in the same context as initial acquisition of associations or in a different context. While enhanced hippocampal activation during extinction learning and recall suggested strengthened context encoding in experimental participants, no behavioral effects upon renewal rates were observed. Atomoxetine also enhanced activation in right iFG during extinction learning, suggesting improved attentional control and response inhibition that contributed to the superior extinction learning performance (Hampshire et al., 2010). Corroborating the previous findings, studies demonstrated that atomoxetine is also involved in processing response inhibition. Single doses of 40 or 60 mg atomoxetine improved inhibitory control together with modulation of prefrontal cortex functioning (Chamberlain et al., 2009). However, Graf et al. (2011) found that a single dose of 80 mg atomoxetine in a sample of healthy male volunteers impaired inhibitory control, which according to the authors might reflect a shift beyond the optimal working range of the noradrenergic system. These studies also found activation in iFG increased by atomoxetine, with one study finding higher activation in right iFG (Chamberlain et al., 2009) and another in bilateral iFG (Graf et al., 2011). Taken together, these results suggest that atomoxetine has a – presumably dose-dependent – capacity to modulate response inhibition in both directions.

In the present study we sought to reveal the role of NA upon learning processes over the complete course of an extinction task, and upon renewal. We assumed that a single dose of the noradrenergic reuptake inhibitor atomoxetine administered prior to acquisition would enhance learning performance and context processing throughout the learning phases, reflecting in fewer errors and increased renewal, combined with strengthened hippocampal and prefrontal activation in atomoxetine participants compared to placebo. Based on our previous findings of enhanced extinction learning after atomoxetine administration we also hypothesized improved performance in the extinction phase of the task, together with increased activation in hippocampus and iFG. Since there is evidence from previous studies that participants with and without a propensity for renewal may respond differentially to pharmacological interventions (e.g., Lissek et al., 2018), we compared subgroups of participants with and without renewal within the treatment groups.

## Materials and Methods

### Participants

Fifty-eight healthy volunteers without a history of neurological disorders or psychiatric illnesses, and without present use of medication (questionnaire, self-report), were recruited by local advertisements and randomly assigned to the treatment (ATO) or control (PLAC) groups. After data acquisition, seven subjects had to be excluded from further data analysis due to inadequate imaging datasets (bad signal or movement artifacts) or missing data. All reported analyses are calculated from the final sample of 51 participants (26 men, 25 women, mean age 26.4 years ± 4.58 sd, range 19–38 years). All participants had normal or corrected-to-normal vision and were right-handed (assessed by means of the Edinburgh Handedness Inventory; Oldfield, 1971). Participants received a monetary compensation (in the amount of 60€). All subjects participated in this study after giving written informed consent. Prior to the experiments, participants received handouts informing them about the fMRI procedure and the pharmacological properties and potential side effects of the NA reuptake inhibitor atomoxetine.

For data analyses, participants were assigned to the groups of (a) participants showing renewal (REN) or (b) not showing renewal (NoREN) based on their performance during the recall phase in trials designed to evoke renewal (i.e., ABA trials with consequence change) by applying *a priori* cut-off values (see Lissek et al., 2015b, 2016, 2017, 2018). All participants who never, or in only a single response, showed renewal (i.e., who had 0–10% renewal responses) were assigned to the NoREN groups (10% renewal was assigned to NoREN to account for a possible single erroneous response). All participants who showed a considerable percentage of renewal responses (30–100% renewal responses) were assigned to the REN groups. In the atomoxetine group, 11 participants were assigned to the REN group and 17 participants were assigned to the NoREN group. In the placebo group, 11 participants were assigned to the REN group and 12 participants were assigned to the NoREN group.

### Predictive Learning Task

The predictive learning task (Üngör and Lachnit, 2006) used in this study is a task for context-related extinction learning without a fear component, suited to reliably evoking a renewal effect by using an ABA design in the experimental condition, contrasted against an AAA design in the control condition. Previous studies already used this task in different versions (e.g., Lissek et al., 2013, 2018; Kinner et al., 2016; Klass et al., 2017). In this task, participants are asked to put themselves in the position of a physician and predict whether various food items served in different restaurants will lead to the aversive consequence of a stomach ache in their patient.

During the initial acquisition phase participants learn to associate a presented food item with a consequence. In each trial a stimulus (photo of a vegetable or a fruit) is presented to the participant in one of two different contexts, which consist of the restaurant names “Zum Krug” (The Mug, 1) and “Altes Stiftshaus” (The Dome, 2) and a frame in either red or blue color. The stimulus in its context is first presented for 3 s, then a question asking whether the patient will develop a stomach ache is superimposed, together with the response options “Yes” or “No.” Response time is 4 s, participants respond by pressing the respective button on an fMRI-ready keyboard (Lumitouch, Photon Control Inc., Canada). After the response, else after expiration of the response time, a feedback with the correct answer is displayed for 2 s, i.e., “The patient has a stomach ache” or “The patient does not have a stomach ache.” The actual response of the participant is not commented upon. The food stimuli are presented in randomized order. The acquisition phase contains 16 different stimuli, 8 stimuli per context. Each stimulus is presented eight times, amounting to a total of 128 trials. Half of the stimuli predict stomach ache, the others predict no stomach ache. The consequence of stomach ache is counterbalanced to appear equally often in both contexts.

During the extinction phase, half of the stimuli from the acquisition phase (eight) are presented again. Of these, one half (four) is presented in the same context as during acquisition (condition AAA – no context change) and the other half (four) in a different context (condition ABA – context change) in randomized order. Within these groups of stimuli a further distinction is being made between actual extinction stimuli (i.e., stimuli for which the consequence of stomach ache changes) and retrieval stimuli (for which the consequence of stomach ache does not change), resulting in each two extinction stimuli and two retrieval stimuli per context. In addition, four new stimuli are introduced during the extinction phase, to balance the design to contain equal numbers of stimuli predicting stomach ache in both contexts, and to investigate new learning in parallel to extinction learning. Therefore, the extinction phase contains a total of 12 different stimuli, 6 per context, with each stimulus being presented eight times, amounting to a total of 96 trials. Again, half of the stimuli predict stomach ache, the others predict no stomach ache, and the consequence of stomach ache is counterbalanced to appear equally often in both contexts. In all other respects, trial design is identical to acquisition.

**FIGURE 1:**
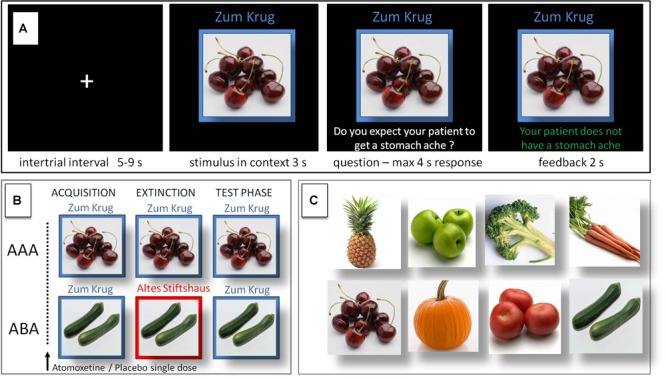
Predictive learning task. **(A)** Single trial sequence. **(B)** Experimental condition with extinction in a novel context (ABA) designed to evoke renewal, and control condition (AAA) with extinction in the identical context. **(C)** Examples of stimuli presented during the task.

During the recall phase, extinction and retrieval stimuli are presented once again in the context of acquisition (five presentations per stimulus), resulting in a total of 40 trials. With the exception that during the recall phase participants receive no feedback with the correct response, trials are identical to those during acquisition. See Table 1 and Figure 1 for an overview of the task design.

**Table 1:** Task design of the predictive learning task (note that the classification of stimuli into extinction, retrieval, and new learning stimuli only applies from the extinction phase on).

Condition		Acquisition		Extinction		Test	
		Context 1	Context 2	Context 1	Context 2	Context 1	Context 2
AAA	Extinction	A+	B+	A-	B-	A?	B?
	Retrieval	C+	D-	C+	D-	C?	D?
	New learning	I-	J-	K+	L+		
		Q-	R+				
ABA	Extinction	E+	F+	F-	E-	E?	F?
	Retrieval	G+	H-	H-	G+	G?	H?
	New learning	M-	N-	P+	O+		
		S-	T+				

*Extinction stimuli were A, B in the AAA condition, and E, F in the ABA condition. Retrieval stimuli (i.e., stimuli that did not change their consequence in the extinction phase) were C, D in the AAA condition and G, H in the ABA condition. New learning stimuli appeared only in the respective learning phase. Overall, + and – signs after the stimuli letters indicate whether the stimulus signaled stomach ache (=CS+) or no stomach ache (=CS-).*

### Procedure

The NA reuptake inhibitor atomoxetine was administered orally in a single dose of 60 mg. Control participants received an identical-looking placebo. After drug administration, participants rested for 90 min. The following fMRI session, in which participants performed the task, covered a time window of about 90–150 min after administration of the drug. Since the phase of peak plasma levels for atomoxetine is assumed to occur 60–120 min after oral ingestion in adults (Sauer et al., 2005; Chamberlain and Robbins, 2013), our task timing provided for peak plasma levels during the acquisition phase of the task and still high plasma levels throughout the following phases. Half-life varies extensively between subjects depending on their metabolism, ranging between 5.2 and 21.6 h (Sauer et al., 2005). Data from the literature suggest that a single dose of 60 mg atomoxetine has smaller effects upon salivary cortisol levels than a natural stressor, e.g., a demonstration lesson of young teachers or a laboratory stressor: The salivary cortisol response induced by a single dose of 60 mg atomoxetine ranged between 3 ng/ml immediately after administration to around 4 ng/ml 1.5–4 h after administration (Chamberlain et al., 2007), while the salivary cortisol response to a demonstration lesson of young teachers (Wolfram et al., 2013) ranged between approx. 4.7 ng/ml immediately before and 4 ng/ml after the test, and the peak response produced by a laboratory stressor (Trier Social Stress Test) ranged between around 5.1 ng/ml in males and 3.4 ng/ml in females, according to a meta-analysis (Liu et al., 2017).

### Imaging Data Acquisition

Functional and structural brain scans were acquired using a whole-body 3T scanner (Philips Achieva 3.0 T X-Series, Philips, Netherlands) with a 32-channel SENSE head coil. Blood-oxygen level dependent (BOLD) contrast images were obtained with a dynamic T2^∗^-weighted gradient echo EPI sequence using SENSE (TR 3200 ms, TE 35ms, flip angle 90°, field of view 224 mm, slice thickness 3.0 mm, voxel size 2.0 × 2.0 × 3.0 mm). We acquired 45 transaxial slices parallel to the anterior commissure–posterior commissure (AC–PC) line which covered the whole brain. High resolution structural brain scans of each participant were acquired using an isotropic T1 TFE sequence (field of view 240 mm, slice thickness 1.0 mm, voxel size 1 × 1 × 1 mm) with 220 transversally oriented slices covering the whole brain. The task was presented to the participants via fMRI-ready LCD-goggles (Visuastim Digital, Resonance Technology Inc., Northridge, CA, United States) connected to a laptop which ran specific software programmed in Matlab (Mathworks, Natick, MA, United States). Responses were given by means of an fMRI-ready keyboard (Lumitouch Response Pad, Photon Control Inc., Canada).

### Imaging Data Analysis

For preprocessing and statistical analysis of fMRI data we used the software Statistical Parametric Mapping (SPM), Version 12 (Wellcome Department of Cognitive Neurology, London, United Kingdom), implemented in Matlab R2017b (Mathworks, Natick, MA, United States). Three dummy scans, during which the BOLD signal reached steady state, preceded the actual data acquisition of each session, thus preprocessing started with the first acquired volume. Preprocessing on single subject level consisted of the following steps: slice timing correction to account for time differences due to multislice image acquisition; realignment of all volumes to the first volume for motion correction; spatial normalization into standard stereotactic coordinates with 2 × 2 × 2 mm^3^ using an EPI template of the Montreal Neurological Institute (MNI) provided by SPM, smoothing with a 6 mm full-width half-maximum (FWHM) kernel, in accordance with the standard SPM procedure. The acceptable limit for head motion was 2 mm for translational movements and 0.5° for rotational movements. If these limits were exceeded in a single volume or across the whole scanning session, the data of the respective participant were excluded from further analysis.

In a first level single subject analysis we calculated activation during acquisition, extinction, and recall phases, contrasted against baseline. We modeled regressors for the onset of each context-cue compound, question, and feedback. All regressors were modeled using distinct stick functions convolved with the canonical hemodynamic response function in the general linear model implemented in SPM, in an event-related design. Contrasts used for the second-level analyses were based on the onset of the image of the context-cue compound at the beginning of a trial, compared to baseline. The contrast images from the single subject analyses were entered into second-level random-effects analyses to compare BOLD activation in the treatment and control groups for acquisition, extinction learning, and recall phases in the experimental (ABA) and control (AAA) conditions. We entered the data into a flexible factorial design containing the factors treatment (ATO and PLAC), renewal propensity (REN and NoREN), as well as different learning conditions for some analyses (e.g., context: identical/different; trial type: extinction, retrieval, new learning). To determine areas where learning-related activation during acquisition and extinction differed between treatments and groups, we used “percent errors” (in acquisition and extinction) as a covariate of interest in the SPM flexible factorial design and calculated contrasts between the subgroups with the same treatment and different response tendencies (ATO REN vs. ATO NoREN, PLAC REN vs. PLAC NoREN) and between the subgroups with the same response tendencies and different treatments (ATO REN vs. PLAC REN, ATO NoREN vs. PLAC NoREN) for acquisition and extinction learning.

We restricted our analyses to our *a priori* regions of interest, that is bilateral medial, ventral and orbital prefrontal cortex, and bilateral hippocampus. For these regions we built anatomical ROIs based on the corresponding anatomical regions defined in the WFU pickatlas toolbox implemented in SPM 12. The prefrontal ROI contained the following AAL atlas regions (Tzourio-Mazoyer et al., 2002): bilateral Frontal_Inf_Oper, Frontal_Inf_Orb, Frontal_Inf_Tri, Frontal Sup_Orb, Frontal_Mid_Orb, Frontal_Med_Orb, and Frontal_Sup_Medial.

In general, imaging results are reported in terms of significance on the whole-brain level with FWE-correction, thresholded at *p* < 0.05 peak level. For results marked with an asterisk (^∗^), small volume correction was applied with FWE-correction, thresholded at *p* < 0.05 peak level. In these cases, the respective small volume always consisted of the complete anatomical ROI (i.e., the ROI of bilateral hippocampus or the ROI of combined bilateral medial, ventral, and orbital prefrontal cortex).

### Behavioral Data Analysis

For all three learning phases, log files were recorded that contained information on response latency, response type, and correctness of response, from which we calculated error rates during acquisition and extinction learning, overall rates as well as specific error rates for the different stimulus types (extinction, retrieval, and new learning stimuli). For calculation of the renewal effect, during the recall phase only responses to stimuli with consequence change (extinction stimuli) were analyzed. The behavioral renewal effect in the predictive learning task is supposed to occur only in the condition ABA, due to the context change introduced during extinction learning. In case of renewal, associations learned during acquisition in context A will reappear in the recall phase which is again performed in context A, while extinction was performed in context B. In contrast, the AAA condition constitutes a control condition for extinction learning, since here all learning phases are performed in an identical context. If extinction learning is successful, responses during the recall phase will reflect the associations learned during extinction. Only if extinction learning is impaired, responses in the AAA recall phase will reflect associations learned during acquisition.

Errors in acquisition and extinction learning were defined as responses stating the incorrect association between the context-cue-compound and the consequence. During the recall phase, a response that referred to the association which was correct during acquisition constituted an error in the AAA condition and a renewal response in the ABA condition. Statistical analyses were performed using the IBM SPSS Statistics for Windows software package, version 23.0 (IBM Corp., Armonk, NY, United States). All results are quoted as mean ± standard error of means (SEM), unless stated otherwise.

For the behavioral analyses in which we compared participants who showed or did not show renewal, ATO, and PLAC, participants were assigned to their respective REN subgroup if they showed at least 30% renewal responses during recall.

Basic behavioral performance in the three learning phases was analyzed by means of ANOVA including the four subgroups of ATO REN, ATO NoREN, PLAC REN, and PLAC NoREN. For significant main effects resulting from an ANOVA we calculated planned contrasts comparing ATO and PLAC/REN and NoREN groups to determine which of the groups differed in their performance. If applicable, for our planned contrasts we applied a modified Bonferroni correction (Keppel, 1991).

## Results

### Participants – Overall Proportion of Participants With Renewal

In each of the two groups (ATO *n* = 28; PLAC *n* = 23), 11 participants, representing 39.29% of participants in the ATO group and 47.83% of participants in the PLAC group, showed a certain degree of renewal. REN and NoREN participants were equally distributed across groups as well as within each group (all subjects: χ^2^ = 0.961, *p* = 0.327; ATO: χ^2^ = 1.286, *p* = 0.257; PLAC: χ^2^ = 0.043, *p* = 0.835). This result indicates that presumably the administration of atomoxetine did not *per se* affect an individual’s general tendency to show renewal – a finding that corresponds to the renewal behavior of atomoxetine-treated participants in a previous study (Lissek et al., 2015a).

### Behavioral Results

#### Acquisition

An univariate ANOVA showed no significant differences in acquisition error rates between the four subgroups ATO REN, ATO NoREN, PLAC REN, and PLAC NoREN (main effect *F*(3) = 0.660, *p* = 0.581). These results demonstrate that the atomoxetine treatment did not affect performance during initial acquisition of the associations between stimulus and outcome (see Table 2).

**Table 2:** Percent errors during acquisition ± standard error of means.

Acquisition	REN	NoREN
ATO	16.69%	18.47%
	±2.82	±2.55
PLAC	13.57%	15.95%
	±2.06	±2.46

#### Extinction

A multivariate ANOVA of extinction conditions showed no significant differences between the four subgroups: ATO REN, ATO NoREN, PLAC REN, and PLAC NoREN in new learning (distractors) and memory retrieval (stimuli without consequence change): [*F*(3) = 0.920, *p* = 0.439, respectively, *F*(3) = 0.450, *p* = 0.718]. However, in extinction trials (with a consequence change in a novel context and in the identical context) there was a significant main effect of subgroup: *F*(3) = 3.497, *p* = 0.023. Planned contrasts revealed that in extinction trials, the ATO REN group differed significantly from the group with the same treatment but with a different response tendency [ATO REN vs. ATO NoREN: *t*(47) = 3.138, *p* = 0.003 two-tailed) and from the group with the same response tendency but without treatment [ATO REN vs. PLAC REN: *t*(47) = 2.403, *p* = 0.020 two-tailed], indicating a selective extinction learning deficit in this group that cannot be explained by response tendency or treatment alone (Table 3). In contrast, performance of the ATO NoREN group did not differ from that of the PLAC REN [*t*(47) = 0.461, *p* = 0.647] and PLAC NoREN [*t*(47) = 0.852, *p* = 0.398 two-tailed]. The comparison of ATO REN with PLAC NoREN showed no significant differences either according to the modified Bonferroni correction for multiple comparisons [ATO REN vs. PLAC NoREN: *t*(47) = 2.051 *p* = 0.046 two-tailed].

**Table 3:** Extinction performance of the four subgroups – percent errors ± SEM.

	Extinction trials	Memory retrieval	New learning
	(ABA&AAA)	(ABA&AAA)	(distractors)
ATO REN	28.41%^∗^	11.08%	11.93%
	±3.19	±2.47	±1.92
ATO NoREN	14.71%^∗^	8.82%	10.11%
	±1.44	±1.52	±1.45
PLAC REN	16.76%^∗^	9.09%	11.65%
	±3.16	±3.76	±1.52
PLAC NoREN	18.23%	6.51%	8.33%
	±3.41	±1.69	±1.29

*^∗^Significant group differences between ATO REN and ATO NoREN, ATO REN and PLAC REN.*

The results indicate an exclusive learning deficit in the ATO REN group in extinction trials, while the group was unimpaired in retrieval of unchanged associations and in learning of new associations.

To analyze the time course of learning for the extinction trials, we grouped the presentations of all extinction stimuli into four sequential blocks and calculated error rates for these blocks separately (Table 4). An ANOVA with repeated measures showed a significant main effect of learning block [*F*(3) = 122.302, *p* = 0.000 and of group *F*(3) = 150.754, *p* = 0.000, but no significant interaction *F*(9) = 1.321, *p* = 0.231]. Bonferroni *post hoc* tests revealed a significant difference between ATO REN and the other groups in the later extinction phases, i.e., in block 3 (ATO REN vs. ATO NoREN *p* = 0.003; ATO REN vs. PLAC NoREN *p* = 0.016) and in block 4 (ATO REN vs. ATO NoREN *p* = 0.001; ATO REN vs. PLAC REN *p* = 0.008; ATO REN vs. PLAC NoREN *p* = 0.001).

**Table 4:** Extinction trials learning performance across four blocks – percent errors per block ± SEM.

	Block 1	Block 2	Block 3	Block 4
ATO REN	56.81%	23.86%	20.45%^∗^	14.77%^∗^
	±3.52	±8.16	±7.59	±6.03
ATO NoREN	50.73%	6.62%	1.47%^∗^	0.0%^∗^
	±4.73	±1.89	±1.00	±0.0
PLAC REN	42.04%	14.77%	6.82%^∗^	3.41%^∗^
	±5.13	±7.11	±3.09	±1.76
PLAC NoREN	57.29%	12.50%	3.12%^∗^	0.0%^∗^
	±9.03	±5.10	±1.63	±0.0

*^∗^Significant group differences between ATO REN and ATO NoREN, ATO REN and PLAC REN.*

#### Recall

The atomoxetine treatment had no effect upon the level of context-related ABA renewal exhibited by the participants: ATO REN and PLAC REN showed a comparable level of ABA renewal: *t*(20) = 0.153, *p* = 0.880, and also the level of AAA errors (i.e., renewal in trials with identical context) did not differ significantly between the REN groups: *t*(20) = 1.953, *p* = 0.065. However, ATO REN and ATO NoREN participants differed significantly with regard to AAA errors [*t*(26) = 2.439, *p* = 0.035], while PLAC REN and PLAC NoREN participants did not [*t*(21) = 0.898, *p* = 0.380) (Table 5).

**Table 5:** Recall behavior in the subgroups – percent ABA renewal responses and AAA errors ± SEM.

Recall phase	ABA renewal	AAA errors
ATO REN	66.36%	26.36%^∗^
	±9.07	±10.81
PLAC REN	64.55%	4.54%
	±7.67	±2.81
ATO NoREN	0.5882%	0.0%^∗^
	±0.58	±0
PLAC NoREN	0.8333%	1.6667%
	±0.83	±1.67

*^∗^Significant group difference between ATO REN and ATO NoREN.*

The results show that the atomoxetine treatment had no effect upon ABA renewal, i.e., ATO REN participants did not respond more frequently with associations correct during acquisition, and extinguished in a novel context (ABA trials), than PLAC REN participants. On the other hand, ATO REN responded more often than their NoREN counterparts with associations correct during acquisition, and extinguished in the identical context (AAA trials).

#### Renewal Ratio

To investigate to what extent the ATO REN participants’ tendency to respond with associations that were correct during acquisition occurred not only in ABA, but also in AAA recall, we calculated their renewal ratio and compared it with the PLAC REN group (for further details, see Lissek et al., 2017). The renewal ratio describes the proportion of renewal responses in the changed context condition ABA compared to the identical context condition AAA. A high value indicates that renewal responses occur predominantly in ABA, and thus tend to be context-driven and not based on impaired learning or recall of extinction associations (value of 1.0 – exclusively ABA renewal; value of 0 – same proportion of ABA renewal and AAA errors; and value of -1.0 – exclusively AAA errors). The mean renewal ratio was significantly lower in ATO REN compared to PLAC REN [*t*(20) = 2.245, *p* = 0.036 two-tailed; mean ATO REN: 0.4882 ± 0.155 SEM, mean PLAC REN: 0.8697 ± 0.069 SEM], indicating that the ATO REN group’s overall recall behavior was less context-driven than that of PLAC REN participants, which means that they showed less context consideration.

**FIGURE 2:**
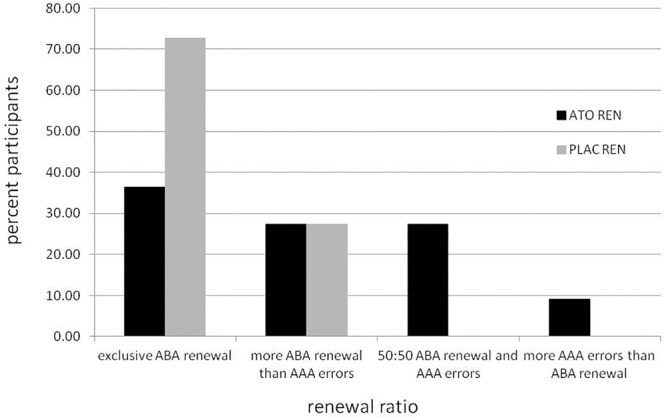
Percentage of participants in the ATO REN and PLAC REN groups exhibiting various combinations of ABA renewal and AAA errors.

Figure 2 illustrates the behavioral tendencies: while 72.73% of PLAC REN participants (8 of 11) showed exclusively ABA renewal, in the ATO REN group there were only 36.36% (4 of 11). More ABA renewal than AAA errors showed 27.27% of ATO REN and 27.27% of PLAC REN (3 of 11 in each group). Only in ATO REN, three subjects (27.27%) showed the same proportion of ABA renewal and AAA errors, and one subject (9.09%) showed more AAA errors than ABA renewal.

### Imaging Results

#### Overview – General Effects of Noradrenergic Stimulation: ATO Compared to PLAC

A first overview over the activation differences associated with noradrenergic stimulation (Table 6) regardless of response tendency revealed significant enhancement of activation in prefrontal regions through all learning phases. While higher activation in ATO in left hippocampus was observed during acquisition, differences in right hippocampus emerged only during extinction and recall.

**Table 6:** Contrasts between ATO and PLAC treatments – differences in learning-related activation in prefrontal and hippocampal ROIs during acquisition, extinction, and recall (two-sample tests, whole brain FWE-corrected *p* < 0.05 on peak level).

Acquisition		ATO > PLAC						PLAC > ATO					
Brain area		MNI coordinates			mm^3^	*t*	*p*	MNI coordinates			mm^3^	*t*	*p*
		X	Y	Z				X	Y	Z			
iFG (frontal operculum)	L	–40	16	32	804	4.78	0.011						
	R	42	20	–16	900	4.83	0.009						
iFG (triangularis) BA 11/47	R	42	44	–2	1164	4.87	0.008						
Lateral OFC BA 10	L	–30	44	26	468	5.87	0.000						
Hippocampus	L	–28	–34	–8	216	3.91	0.043						
	R												

**Extinction**		**ATO > PLAC**						**PLAC > ATO**					
**Brain area**		**MNI coordinates**			**mm^3^**	***t***	***p***	**MNI coordinates**			**mm^3^**	***t***	***p***
		**X**	**Y**	**Z**				**X**	**Y**	**Z**			

iFG (frontal operculum)	L												
	R	–52	16	34	1128	4.49	0.037						
vmPFC BA 10	L	–4	54	26	288	5.14	0.002						
vmPFC BA 10		–10	50	2	1104	4.75	0.013						
Middle frontal g. BA 10 OFC		–32	46	28	1068	5.07	0.003						
iFG BA 45/47		–38	26	2	720	4.98	0.005						
iFG BA 47	R	22	10	–18	144	4.78	0.011						
Lateral OFC BA 10		34	50	4	264	4.86	0.008						
Middle/sup. frontal gyrus BA10		30	52	26	876	4.42	0.050						
Hippocampus	L												
	R	22	–20	–16	1068	4.78	0.002	18	–34	4	84	5.52	0.000

**Recall**		**ATO > PLAC**						**PLAC > ATO**					
**Brain area**		**MNI coordinates**			**mm^3^**	***t***	***p***	**MNI coordinates**			**mm^3^**	***t***	***p***
		**X**	**Y**	**Z**				**X**	**Y**	**Z**			

iFG (frontal operculum)	L	–38	10	28	612	4.60	0.033						
	R												
	L												
Lateral OFC BA 10	R	44	44	16	180	4.70	0.022						
Hippocampus	L												
	R	20	–36	0	204	4.17	0.027						

Subsequently, more specific analyses indicated that some of the differences between ATO and PLAC were presumably masked in this first overview, due to the divergent activation patterns in the ATO subgroups, with ATO REN showing reduced activation relative to PLAC in various regions.

#### Differential Activation in the Treatment Subgroups During *Acquisition*

**FIGURE 3:**
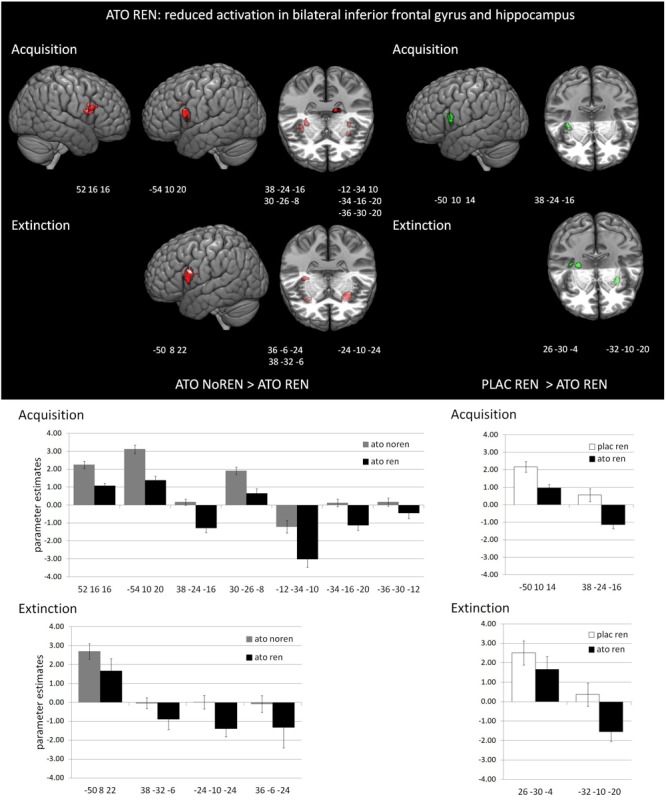
Reduced activation in ATO REN compared to ATO NoREN and PLAC REN (contrasts ATO NoREN > ATO REN; PLAC REN > ATO REN, SVC *p* < 0.05 FWE-corrected). The bar graphs represent the parameter estimates for the subgroups regarding the differentially activated regions: relative to the other groups, ATO REN shows a less prominent activation increase in iFG, and predominantly deactivation relative to baseline in hippocampus.

**FIGURE 4:**
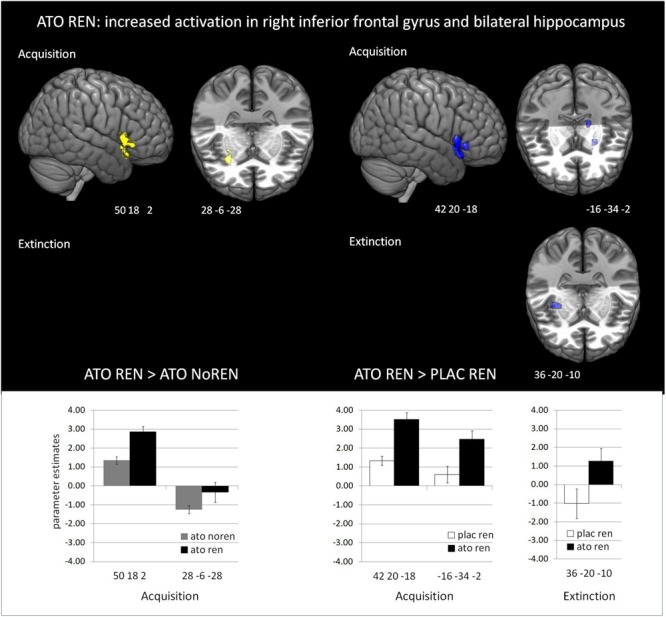
Increased activation in ATO REN compared to ATO NoREN and PLAC REN (contrasts ATO REN > ATO NoREN; ATO REN > PLAC REN, SVC *p* < 0.05 FWE-corrected). The bar graphs represent the parameter estimates for the subgroups regarding the differentially activated regions: ATO REN predominantly shows a stronger increase of BOLD activation relative to baseline than the other groups.

During acquisition, learning-related activation in ATO participants differed not only from PLAC participants, but also between the ATO subgroups: ATO NoREN participants showed higher activation relative to ATO REN and to PLAC NoREN in largely the same regions: in bilateral iFG and bilateral hippocampus. This pattern of stronger activation appears to be related to the combination of treatment and response tendency, since it was present compared to untreated participants with the same response tendency, but also compared to participants with the same treatment, but a different response tendency. In contrast, ATO REN participants showed increased activation relative to ATO NoREN in right iFG and right anterior hippocampus. Higher activation compared to PLAC REN was found also in right iFG and left posterior hippocampus. Activation in ATO REN was reduced relative to ATO NoREN and PLAC REN in left iFG and in right posterior hippocampus. However, reduction of activation was much stronger and encompassed larger regions compared to ATO NoREN – indicating that the noradrenergic stimulation had differential effects upon participants with different response tendencies. The contrasts between PLAC NoREN and PLAC REN or ATO NoREN showed no significantly differentially activated regions (see Table 7 and Figure 3, 4).

**Table 7:** Contrasts between treatments/groups showing differences in learning-related activation in hippocampus and prefrontal ROIs during acquisition [two-sample tests, whole brain FWE-corrected *p* < 0.05 on peak level (^∗^ with small volume correction)].

Acquisition		ATO NoREN > ATO REN						ATO REN > ATO NoREN					
Brain area		MNI coordinates			mm^3^	*t*	*p*	MNI coordinates			mm^3^	*t*	*p*
		X	Y	Z				X	Y	Z			
iFG (frontal operculum)	L	–54	10	20	3900	6.05	0.000						
	R	52	16	16	1464	5.29	0.007	50	18	2	3288	7.22	0.000
Hippocampus	L	–12	–34	10	384	4.44	0.007^∗^						
		–34	–16	–20	240	4.12	0.000^∗^						
		–36	–30	–12	204	4.08	0.015^∗^						
	R	38	–24	–16	408	5.81	0.001	28	–6	–28	600	4.33	0.011^∗^
		30	–26	–8	348	4.46	0.007^∗^						

**Acquisition**		**PLAC REN > ATO REN**						**ATO REN > PLAC REN**					
**Brain area**		**MNI coordinates**			**mm^3^**	***t***	***p***	**MNI coordinates**			**mm^3^**	***t***	***p***
		**X**	**Y**	**Z**				**X**	**Y**	**Z**			

iFG (frontal operculum)	L	–50	10	14	3096	5.40	0.006						
	R							42	20	–18	1548	4.68	0.017^∗^
													
Hippocampus	L							–16	–34	–2	216	4.21	0.017^∗^
	R	38	–24	–16	588	5.25	0.010						

**Acquisition**		**PLAC REN > PLAC NoREN**						**PLAC NoREN > PLAC REN**					
**Brain area**		**MNI coordinates**			**mm^3^**	***t***	***p***	**MNI coordinates**			**mm^3^**	***t***	***p***
		**X**	**Y**	**Z**				**X**	**Y**	**Z**			

iFG (frontal operculum)	L												
	R												
Hippocampus	L												
	R	36	–22	–12	1272	4.80	0.002^∗^						

**Acquisition**		**ATO NoREN > PLAC NoREN**						**PLAC NoREN > ATO NoREN**					
**Brain area**		**MNI coordinates**			**mm^3^**	***t***	***p***	**MNI coordinates**			**mm^3^**	***t***	***p***
		**X**	**Y**	**Z**				**X**	**Y**	**Z**			

iFG (frontal operculum)	L	–40	16	32	3612	5.92	0.001						
	R	54	16	26	4092	5.05	0.022						
Middle frontal gyrus	L	–30	46	26	1092	5.67	0.002						
	R	28	52	28	456	5.66	0.002						
Hippocampus	L	–16	–10	–12	432	5.27	0.010						
		–30	–32	–12	468	4.48	0.006^∗^						
	R	38	–24	–12	1824	4.75	0.002^∗^						

#### Differential Activation in the Treatment Subgroups During *Extinction*

During extinction learning, ATO REN participants’ activation was reduced compared to ATO NoREN in left iFG and bilateral hippocampus. Compared to PLAC REN, activation was reduced in right posterior hippocampus and left anterior hippocampus, but increased in a region in right mid-hippocampus and right iFG. The remaining contrasts between PLAC NoREN and PLAC REN as well as PLAC NoREN and ATO NoREN yielded no significantly differentially activated regions (see Table 8 and Figure 3, 4).

**Table 8:** Contrasts between treatments/groups showing differences in learning-related activation in hippocampus and prefrontal ROIs during extinction learning [two-sample tests, whole brain FWE-corrected *p* < 0.05 on peak level (^∗^ with small volume correction)].

Extinction		ATO NoREN > ATO REN						ATO REN > ATO NoREN					
Brain area		MNI coordinates			mm^3^	*t*	*p*	MNI coordinates			mm^3^	*t*	*p*
		X	Y	Z				X	Y	Z			
iFG (frontal operculum)	L	–50	8	22	4104	5.67	0.002						
Hippocampus	L	–24	–10	–24	1248	4.34	0.010^∗^						
	R	36	–6	–24	456	4.36	0.010^∗^						
		38	–32	–6	240	3.84	0.052^∗^						

**Extinction**		**ATO REN > PLAC REN**						**PLAC REN > ATO REN**					
**Brain area**		**MNI coordinates**			**mm^3^**	***t***	***p***	**MNI coordinates**			**mm^3^**	***t***	***p***
		**X**	**Y**	**Z**				**X**	**Y**	**Z**			

iFG (frontal operculum)	L												
	R	40	28	–4	1284	5.05	0.023						
Hippocampus	L							–32	–10	–20	684	3.94	0.039^∗^
	R	36	–20	–10	324	4.11	0.022^∗^	26	–30	–4	300	4.11	0.023^∗^

#### Differential Activation in ATO REN, ATO NoREN, and PLAC Groups During Retrieval Trials in the Extinction Phase Relating to Associative Strength

To investigate whether associative strength differed between groups, we analyzed BOLD activation in retrieval trials, in which only the recall of previously learned associations was required. We calculated contrasts between the groups, specifically focusing on areas that were found sensitive to associative strength of learned stimuli, i.e., left anterior and mid-ventrolateral PFC [BA areas 47 and 12; BA area 45 (see Badre et al., 2005)]. Studies showed that these areas exhibited higher activation when the associative strength between two stimuli was weak (Wagner et al., 2001; Badre et al., 2005; Danker et al., 2008). Using the WFU pickatlas tool, we devised an anatomical ROI mask comprising these areas and analyzed activation differences between ATO and PLAC subgroups. Results demonstrate that ATO REN showed consistently reduced activation in left-hemispheric anterior and mid-ventrolateral PFC, compared to ATO NoREN (MNI coordinates -54 10 14, 236 voxel, *t* = 5.36, *p* = 0.007; -44 0 6, 26 voxel, *t* = 4.18, *p* = 0.050^∗^) and to PLAC REN (-34 20 -10, 216 voxel, *t* = 4.71, *p* = 0.014). In addition, ATO NoREN showed higher activation than PLAC groups in this region (MNI coordinates -58 14 20, 241 voxel, *t* = 4.30, *p* = 0.034). This reduced activation in ATO REN suggests higher associative strength of their associations learned during acquisition.

#### Differential Activation in ATO REN, ATO NoREN, and PLAC Groups During *Recall*

**FIGURE 5:**
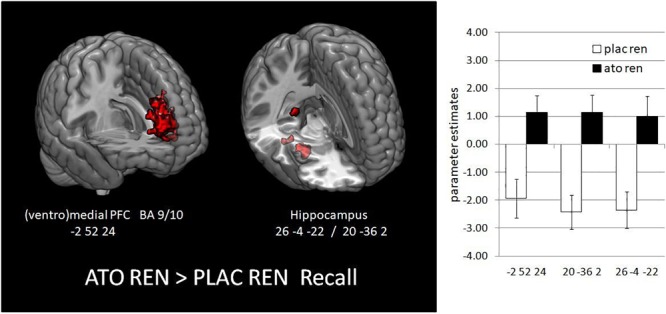
Activation increase in ATO REN compared to PLAC REN during recall in vmPFC and in hippocampus (contrast ATO REN > PLAC REN, SVC *p* < 0.05 FWE-corrected). The bar graphs represent the parameter estimates for the subgroups in the differentially activated regions.

During recall, ATO REN showed increased activation compared to PLAC REN in vmPFC and lateral OFC (BA 10) as well as in right anterior and posterior hippocampus. In particular, PLAC REN showed deactivations in these regions, while activation in ATO REN was enhanced relative to baseline. Also relative to ATO NoREN, ATO REN showed increased activation in right posterior hippocampus. PLAC NoREN and PLAC REN did not differ in their prefrontal and hippocampal activation during recall. Also in the contrasts of ATO NoREN with PLAC NoREN we observed no differential activation (see Table 9 and Figure 5).

**Table 9:** Contrasts between treatments/groups showing differences in activation in hippocampus and prefrontal ROIs during recall [two-sample tests, whole brain FWE-corrected *p* < 0.05 on peak level (^∗^ with small volume correction)].

Recall		ATO NoREN > ATO REN						ATO REN > ATO NoREN					
Brain area		MNI coordinates			mm^3^	*t*	*p*	MNI coordinates			mm^3^	*t*	*p*
		X	Y	Z				X	Y	Z			
iFG (frontal operculum)	L												
	R												
Hippocampus	L							–20	–28	–6	1164	6.47	0.000
	R												

**Recall**		**ATO REN > PLAC REN**						**PLAC REN > ATO REN**					
**Brain area**		**MNI coordinates**			**mm^3^**	***t***	***p***	**MNI coordinates**			**mm^3^**	***t***	***p***
		**X**	**Y**	**Z**				**X**	**Y**	**Z**			

iFG (frontal operculum)	L												
	R												
(Ventro)medial PFC BA 10/9	L	–2	52	24	9336	5.72	0.001^∗^						
Lateral OFC BA10	R	44	46	16	216	4.74	0.037^∗^						
Hippocampus	L												
	R	20	–36	2	252	4.48	0.010^∗^						
		26	–4	–22	768	4.37	0.014^∗^						

## Discussion

The noradrenergic stimulation had differential effects upon the atomoxetine-treated subgroup with a propensity for renewal (ATO REN) compared to the subgroup without a propensity for renewal (ATO NoREN) and the control subgroup who showed renewal (PLAC REN).

In the ATO REN group, noradrenergic stimulation impaired extinction learning, presumably due to formation of more stable associations and disturbed response inhibition, and reduced context-dependent renewal. Accordingly, BOLD activation differences in ATO REN, compared to the other groups, indicated impairments of learning-relevant processes. Modulated activation in bilateral iFG affected response inhibition and/or decision processes, while altered activation in bilateral hippocampus had detrimental effects upon context processing.

### Atomoxetine Impaired Extinction Learning and Reduced Context-Dependent Renewal in ATO REN

#### Impaired Extinction Learning in ATO REN

Contrary to our hypothesis, a single dose of 60 mg atomoxetine prior to acquisition impaired later extinction learning in ATO REN, and had no beneficial effect upon ATO NoREN extinction, compared to PLAC. Since in a previous study (Lissek et al., 2015a), atomoxetine prior to the extinction phase improved extinction learning in the ATO group, the extinction performance observed in the present study is presumably related to the changed timepoint of administration. However, the treatment had no significant effect upon the groups’ error rates during acquisition proper. Presumably the relatively easy task of acquiring stimulus–consequence associations did not lend itself to revealing treatment-related differences, which resulted in a ceiling effect. This assumption is supported by the fact that REN and NoREN participants’ acquisition performance within the ATO and PLAC groups did not differ. This finding corresponds to previous studies using the same task (e.g., Lissek et al., 2013, 2015a) and further suggests that atomoxetine did not differentially modulate processing in REN and NoREN groups during acquisition. In comparison to initial learning, the extinction task represented a higher level of difficulty, requiring additional processes of response inhibition and selection. This higher level of difficulty combined with the atomoxetine treatment and the propensity for renewal presumably caused the observed selective extinction deficit of the ATO REN group.

Since NA has enhancing effects upon memory, it can be speculated that a noradrenergic enhancement during acquisition – that did not reflect in acquisition error rates – manifested itself only during extinction. If the noradrenergic stimulation contributed to establishing a more robust memory of stimulus–consequence associations during acquisition, the demands upon extinguishing these obsolete associations during extinction were more pronounced in the treatment group. In this case, a beneficial effect of atomoxetine upon extinction learning (as observed in our previous study (Lissek et al., 2015a) was presumably masked by the increased task difficulty for the ATO subjects, leading to an extinction learning performance in the ATO NoREN group statistically not different from the PLAC groups. For the ATO REN participants, task difficulty during extinction learning was probably even higher, since during acquisition they had integrated the context in their associations (see Lissek et al., 2016), resulting in context–cue–consequence associations with high associative strength that presumably were particularly resistant to change. During the extinction phase, this change resistance reflected in the ATO REN group’s higher error rates. Studies on modulation of associative strength and noradrenergic enhancement of memory support these assumptions. Brain regions particularly sensitive to associative strength in retrieval are left anterior and mid-ventrolateral PFC, corresponding roughly to BA 47 and BA 12 on the one hand and BA 45 on the other (Nyhus and Badre, 2015). Experiments that manipulated associative strength (Wagner et al., 2001; Badre et al., 2005; Danker et al., 2008)consistently found higher activation in these regions in weak compared to robust associative strength. Correspondingly, our analysis of these regions showed for ATO REN reduced BOLD activation in retrieval trials, suggesting that their associative strength was higher. Consequently, the associations ATO REN had established during acquisition were presumably harder to extinguish. Such an associative-strength assumption can also reconcile the previous findings (Lissek et al., 2015a) with the present results: in our previous study, noradrenergic stimulation did not affect acquisition associations, since atomoxetine was administered after acquisition; therefore, the associative strength of acquisition associations was probably similar in the experimental and the placebo group. During extinction, however, only the atomoxetine-treated group benefitted from a noradrenergic enhancement which enabled them to learn faster.

Ample evidence for such enhancing effects of NA upon memory formation is found in the literature: NA is a key neuromodulator that can regulate the degree of learning and memory (Roozendaal et al., 2009; Joëls et al., 2011; Díaz-Mataix et al., 2017). A mechanism for enhancement of memory formation is lowering the threshold for LTP (Hu et al., 2007). Accordingly, amplification effects of NA have been observed in aversive Pavlovian conditioning (Díaz-Mataix et al., 2017), spatial working memory (Hvoslef-Eide et al., 2015), and reversal learning (Glennon et al., 2018), as well as for prefrontal cognitive functions in monkeys (Gamo et al., 2010). A recent model explained noradrenergic enhancement for high priority information by an additive effect of glutamate and NA, whereby increases in NA enable further enhancement of already highly activated, prioritized mental representations (Mather et al., 2016). In the present study, the context–cue–associations formed by ATO REN might have constituted such prioritized representations. Together, the findings on beneficial noradrenergic effects upon memory provide additional evidence that, in our study, memory formation of initial learning was enhanced by the atomoxetine treatment.

In addition, the noradrenergic stimulation probably also affected response inhibition, since atomoxetine can have dose-dependent effects upon response inhibition. Single doses of 40 or 60 mg (Chamberlain et al., 2009) enhanced inhibition, while a study using 80 mg (Graf et al., 2011) found impairing effects of atomoxetine upon response inhibition, arguing that they might reflect a shift beyond the optimal working range of the noradrenergic system. Since our single dose of 60 mg atomoxetine had enhanced extinction learning before, a dose-dependent effect appears rather unlikely. However, attentional arousal induced by the stimuli (context-cue-compounds) might have been overall higher in renewal participants, thus generating a higher level of noradrenergic activation from the start. In this case, an additional dose of atomoxetine might have brought about a shift beyond the optimal working range of the noradrenergic system in the ATO REN subjects (as proposed by Graf et al., 2011), which contributed to the observed learning impairments.

#### Reduced Context-Dependent Renewal in ATO REN

During recall, the ABA renewal rates of the REN groups did not differ, which is in line with the findings of our previous study. Thus, it appears that noradrenergic stimulation, whether prior to acquisition or prior to extinction, does not affect the overall renewal level after extinction in a different context. However, the comparably high AAA error level in ATO REN suggests that their renewal responses were partially not associated with the context, an assumption that also reflects in their renewal ratio, which was significantly lower than in the PLAC REN group. In summary, it appears that the overall recall performance of the ATO REN group was based partially on context-independent recall of acquisition associations, a behavior which in turn was largely caused by the group’s impaired extinction learning not only for ABA but also for AAA trials.

### Noradrenergic Stimulation Modulated BOLD Activation in Prefrontal Cortex and Hippocampus Differentially in ATO REN and NoREN

In all ATO participants analyzed together, the noradrenergic stimulation increased BOLD activation in various regions in prefrontal cortex and in hippocampus during all three task phases, compared to PLAC, which is in line with the results of our previous atomoxetine study (Lissek et al., 2015a). However, BOLD activation also differed between the ATO REN and NoREN subgroups in these regions, which, in view of their identical treatment, was probably associated with their preferred processing strategies. During acquisition and extinction learning, ATO REN showed differential activation in areas relevant for context processing and executive control that presumably influenced the respective learning processes in a manner that contributed to the observed learning performance: reduced activation in left iFG, as well as in several regions in bilateral hippocampus, relative to both ATO NoREN and PLAC REN; and increased activation in right iFG during acquisition, relative to ATO NoREN and during acquisition and extinction relative to PLAC REN, as well as increased activation, in a few hippocampal regions relative to ATO NoREN and PLAC REN during acquisition, and relative to PLAC REN during extinction.

#### Atomoxetine Modulated Bilateral Inferior Frontal Gyrus Activation in ATO Groups

Recent studies have implicated iFG in response inhibition, an executive control process important for extinction learning (Bouton et al., 2016). FMRI studies showed that particularly in right iFG the BOLD signal increased during response inhibition (Menon et al., 2001; Rubia et al., 2003; Aron et al., 2014). Right iFG is also involved in attentional control (Hampshire et al., 2010). Importantly, it was found that atomoxetine increases right iFG activation during inhibitory control processes (Chamberlain et al., 2009). These results correspond to our findings of overall increased right iFG activation in the ATO subgroups during acquisition and extinction – an activation which presumably indicates enhanced processing of stimuli relevant for adequate response selection. However, ATO REN and ATO NoREN exhibited different activation peaks within right iFG. Also relative to PLAC REN, ATO REN exhibited increased activation in right iFG. Due to the ability of atomoxetine to increase right iFG activity during inhibitory control combined with improved response inhibition (Chamberlain et al., 2009), this increased activation appears to signal pronounced inhibition of responses that have proved incorrect before – a processing mode which may be related to the formation of more robust associations. However, during extinction, when response inhibition is more relevant, right iFG activation did not differ between the ATO subgroups, and thus, presumably, neither its contribution to response inhibition. Therefore, impaired right iFG response inhibition presumably was not a crucial factor for the ATO REN group’s extinction deficit.

During acquisition and extinction, we also observed reduced left iFG activation in ATO REN relative to ATO NoREN, and relative to PLAC REN during acquisition. Left iFG appears to have a role in response inhibition too, as shown in studies with human lesion patients performing a Go–NoGo task (Swick et al., 2008), one of which attributed the observed deficit to decisional rather than inhibitory processes (Arbula et al., 2017). A different hypothesis posits that left iFG is part of a network of frontal lobe subsystems that detect and resolve incompatible stimulus representations (Novick, 2005), suggesting that left iFG has a role in conflict resolution. Thus, the overall downregulation of activity in left iFG in ATO REN compared to both ATO NoREN and PLAC REN presumably contributed to their impairment during extinction learning, by affecting response inhibition and/or decision or conflict resolution processes.

#### Atomoxetine Modulated Hippocampus Activation in ATO Groups

Overall, in the ATO groups, hippocampal activation was strengthened by the noradrenergic stimulation, again in line with our previous atomoxetine study (Lissek et al., 2015a). However, in the present study atomoxetine affected hippocampal activation during acquisition and extinction differentially in ATO REN and ATO NoREN participants: While hippocampal BOLD activation in ATO NoREN was characterized predominantly by a small increase relative to baseline, in ATO REN several hippocampal regions showed a deactivation relative to baseline, compared to ATO NoREN, and also to PLAC REN. Only in a few hippocampal regions did ATO REN exhibit an increase relative to baseline.

Actually, NA can evoke a bidirectional modulation of hippocampal activity, as demonstrated by an *in vitro* study in rat hippocampal slices (Madison and Nicoll, 1986), where an initial hyperpolarization was followed by depolarization. Another potential mechanism causing hippocampal deactivation is atomoxetine-induced voltage-dependent NMDA receptor blockade (Ludolph et al., 2010). For the opposing activation patterns of ATO REN and NoREN observed in the present study, it can therefore be assumed that this effect was related to the participants’ preferred processing strategies that presumably recruited differentially interconnected networks, in which noradrenergic activation could produce distinct, opposite consequences.

The most consistent hippocampal deactivation in ATO REN compared to ATO NoREN and PLAC REN was observed in bilateral posterior hippocampus, particularly in the right-hemispheric portion. An fMRI study investigating the contributions of anterior and posterior hippocampus for (visuospatial) encoding and retrieval in humans (Duarte et al., 2014) found that the encoding process elicited BOLD activation in bilateral posterior hippocampus, predominantly in the right hemisphere, with concurrent deactivation of bilateral anterior hippocampus. Retrieval elicited BOLD activation in right posterior hippocampus and deactivation in bilateral anterior hippocampus. Correspondingly, in previous studies hippocampal activation during encoding and recall was predominantly located in posterior regions (e.g., Lissek et al., 2015a, 2017, 2018; Golisch et al., 2017), suggesting that generally, encoding and recall in contextual associative learning recruit similar regions as the above mentioned visuospatial task. So in the light of the findings by Duarte et al., the hippocampal activation pattern in ATO REN lacked the right posterior component important for encoding and retrieval, which presumably modulated or weakened these processes and thus impaired extinction context encoding in a way that had an adverse impact upon response selection in recall. Accordingly, our previous studies suggest that bilateral hippocampal activity plays an important role for context encoding both during initial learning and extinction: in a study evaluating hippocampal processing during acquisition in untreated participants (Lissek et al., 2016), bilateral hippocampus was activated in the group that showed renewal. Also during extinction, bilateral hippocampal activation was higher in REN than NoREN participants (Lissek et al., 2013). These findings were corroborated by further pharmacological studies, where drug-modulated hippocampal activation in REN groups was associated with extinction learning performance (Lissek et al., 2015a, 2017, 2018). Thus, strong bilateral hippocampal recruitment conceivably constitutes a necessary prerequisite for successful context-related extinction learning, so that in the present study, the overall reduction in hippocampal activation of ATO REN during extinction presumably contributed to the learning deficit.

Besides the dominant hippocampal deactivation compared to the other groups, ATO REN showed increased activation in left posterior hippocampus during acquisition and right posterior hippocampus during extinction, relative to PLAC REN. Thus the REN groups’ hippocampal activation peaks were located in different positions, which may have been incidental to processing outcomes.

#### Increased Activation in Hippocampus and Prefrontal Cortex in ATO REN During Recall

In the recall phase, we observed a departure from the pattern prevalent during acquisition and extinction: ATO REN showed significantly higher activation in right posterior and anterior hippocampus compared to PLAC REN, and no regions of reduced activation relative to ATO NoREN and PLAC REN.

In right anterior and posterior hippocampus activation increased from baseline in ATO REN, opposed to a deactivation in PLAC REN. Thus, the pattern of anterior deactivation and posterior higher activation supposed to characterize retrieval (Duarte et al., 2014) was not present. Thus, the retrieval of previously encoded information may have been modulated in ATO REN in a way that contributed to the group’s reduced context-dependent renewal. Even though both REN groups did not differ in their level of ABA renewal, the higher level of AAA errors in ATO REN indicated a retrieval mode that favored associations acquired during initial learning at the cost of associations established during extinction, regardless of context. This behavior of ATO REN may have resulted from a potential additive effect of an internal processing tendency requiring pronounced hippocampal recruitment, and noradrenergic stimulation – a combination that presumably caused a shift beyond the optimal working range of the noradrenergic system (see Graf et al., 2011). Another potential contributor to this behavior may have been compromised context encoding during extinction learning, so that even prominent hippocampal activation during recall did not deliver proper context information at all times. While activation in bilateral iFG did not differ between the groups, ATO REN showed increased activation – opposed to the deactivation in PLAC REN – in a large cluster in bilateral medial PFC (medial BA 10 and BA9), ranging from vmPFC to orbitofrontal ventral regions. Activation in a more ventrally located portion of vmPFC during recall has previously been found to be stronger in renewal participants during ABA trials, and thus has been linked to ABA renewal (Lissek et al., 2013). However, in the present study, both REN groups showed a similar level of ABA renewal, despite their divergent medial prefrontal activation. Since it has been proposed that during recall, vmPFC retrieves context information provided by hippocampus (Lissek et al., 2013) and retrieval of context information appeared partially compromised in ATO REN in the present study, it can be speculated that also here we observe an adverse interactive effect of response tendency-based pre-recruitment of brain regions, and stimulation of the noradrenergic system by atomoxetine, which together may have caused prominent activation of this prefrontal region without a behavioral performance benefit for proper response selection.

### Limitations of the Study

Since this study used a cognitive associative learning task instead of a fear extinction task, respectively, an aversive Pavlovian conditioning procedure, the present findings on atomoxetine effects cannot be readily transferred for clinical therapeutic purposes. Of note, various studies have demonstrated that the extinction network recruited during our predictive learning task is similar to that recruited in fear extinction studies, with the exception of amygdala participation (e.g., Lissek et al., 2013, 2015a,b, 2016, 2018). Therefore, the results can provide interesting hints for future research using a fear extinction task.

## Summary/Conclusion

In this study we demonstrate for the first time diverging effects of noradrenergic stimulation upon extinction learning performance and corresponding brain activation in healthy volunteers with opposing context processing tendencies. The NA reuptake inhibitor atomoxetine, administered prior to acquisition, significantly impaired extinction learning exclusively in participants who showed contextual renewal during recall (ATO REN). In this group, increased activation in right iFG may have supported the formation of robust, change-resistant associations during acquisition, while pronounced deactivation in bilateral hippocampus and reduced activation in left iFG presumably impaired context encoding and response inhibition during extinction and so contributed to the extinction learning deficit. During recall, ATO REN showed partially context-independent retrieval, resulting from a high level of AAA renewal responses, together with an ABA renewal level comparable to PLAC REN. Thus, context retrieval and response selection were at least partially compromised by deficient input, despite higher activation in medial PFC and right hippocampus. In summary, the opposing responses to identical noradrenergic stimulation of participants with and without a propensity for renewal were presumably related to their preferred processing strategies which recruited differentially interconnected networks responding to noradrenergic stimulation in different ways. In ATO REN, the stimulation presumably caused a shift beyond the optimal working range of the noradrenergic system, resulting from potential additive effects of the group’s specific internal processing tendencies which pre-activated the noradrenergic system, and the administration of atomoxetine.

## Ethics Statement

All subjects participated in this study after giving written informed consent. The study protocol was approved by the local Ethics Committee of the Ruhr-University Bochum. The study conforms to the Code of Ethics of the World Medical Association (Declaration of Helsinki).

## Author Contributions

MT and SL developed the study conception and the design. SL interpreted the data, performed data analyses, and wrote the manuscript. AK acquired the data and performed statistics. AK and MT made critical revisions. All authors contributed to and have approved the final manuscript.

## Conflict of Interest Statement

The authors declare that the research was conducted in the absence of any commercial or financial relationships that could be construed as a potential conflict of interest.
